# Tibolone Improves Motor Recovery and Regulates Neuroinflammation and Gliosis in a Model of Traumatic Spinal Cord Injury

**DOI:** 10.3390/ijms26178327

**Published:** 2025-08-27

**Authors:** Ximena Freyermuth-Trujillo, Stephanie Sánchez-Torres, Carlos E. Orozco-Barrios, Hermelinda Salgado-Ceballos, Julia J. Segura-Uribe, Christian Guerra-Araiza, Ángel León-Cholula, Isabel Arrieta-Cruz, Julio Morán, Angélica Coyoy-Salgado

**Affiliations:** 1 Unidad de Investigación Médica en Farmacología, Hospital de Especialidades Dr. Bernardo Sepúlveda, Centro Médico Nacional Siglo XXI, Instituto Mexicano del Seguro Social, Mexico City 06720, Mexicochristianguerra2001@gmail.com (C.G.-A.);; 2 Posgrado en Ciencias Biológicas, Universidad Nacional Autónoma de México, Mexico City 04510, Mexico; 3 SECIHTI-Unidad de Investigación Médica en Enfermedades Neurológicas, Hospital de Especialidades, Centro Médico Nacional Siglo XXI, Instituto Mexicano del Seguro Social, Mexico City 06720, Mexico; 4 Unidad de Investigación Médica en Enfermedades Neurológicas, Hospital de Especialidades Dr. Bernardo Sepúlveda, Centro Médico Nacional Siglo XXI, Instituto Mexicano del Seguro Social, Mexico City 06720, Mexico; 5 Departamento de Investigación Farmacoepidemiológica, Hospital Infantil de México Federico Gómez, Secretaría de Salud, Mexico City 06720, Mexico; drjuseur.farmaepid@gmail.com; 6 Departamento de Investigación Básica, División de Investigación, Instituto Nacional de Geriatría, Secretaría de Salud, Mexico City 10200, Mexico; 7 División de Neurociencias, Instituto de Fisiología Celular, Universidad Nacional Autónoma de México, Mexico City 04510, Mexico; jmoran@ifc.unam.mx

**Keywords:** neuroprotection, sexual hormones, inflammation, motor function recovery, central nervous system, gliosis

## Abstract

Spinal cord injury (SCI) results in significant motor, sensory, and autonomic dysfunction. The pathophysiology of SCI develops during the primary and secondary phases. Inflammation contributes to the secondary phase through the non-specific activation of the innate immune response. Glial scar formation (gliosis), a reactive cellular mechanism facilitated by astrocytes, also occurs during this phase. Synthetic steroids such as tibolone (Tib) have been proposed as a treatment for SCI since they exert neuroprotective effects in various models of central nervous system (CNS) injury. We studied the effect of Tib on locomotor functional recovery and the regulation of neuroinflammation and gliosis in an SCI model. We performed an SCI at the thoracic vertebrae nine in male Sprague Dawley rats. The animals received daily doses of Tib (1 or 2.5 mg per kg of body weight) administered orally. We quantified pro- and anti-inflammatory cytokine levels at the injury site and determined motor recovery using the Basso, Beattie, and Bresnahan (BBB) scale. Finally, we investigated the effect of Tib on the expression of glial fibrillary acidic protein (GFAP) and ionized calcium-binding adaptor molecule 1 (Iba-1), two markers of gliosis, using an immunohistochemistry assay. Our findings showed that Tib regulated pro- and anti-inflammatory cytokine levels at 3 h and 3, 7, and 14 days post-SCI. Furthermore, Tib administered orally for 15 days reduced gliosis markers and favored tissue preservation and motor function recovery after SCI.

## 1. Introduction

Spinal cord injury (SCI) is a complex health issue that can cause paraplegia and quadriplegia. About 2.5 million people worldwide are affected by SCI, with an annual rise of 130,000 new cases [1,2]. The lifetime medical costs for an SCI patient range from USD 500,000 to USD 2 million [3]. SCI often results from motor vehicle accidents, falls, acts of violence, and sports-related events [4]. Data show that SCI is more common among young adult males, with the male-to-female ratio increasing over time. In developing countries, the main causes of traumatic SCI are motor vehicle crashes and falls [5,6].

Inflammation occurs during the secondary phase of SCI [7,8]. This process is driven by multiple cell types and a variety of inflammatory cytokines, including tumor necrosis factor-alpha (TNF-α), interleukin-1β (IL-1β), interleukin-6 (IL-6), interleukin-1α (IL-1α), interferon-gamma (IFN-γ), and granulocyte-macrophage colony-stimulating factor (GM-CSF), among others. The extensive infiltration of immune cells leads to neural degeneration [9,10]. These immune cells are directed to the lesion site from the periphery by cytokines and chemokines released by microglia, astrocytes, and peripherally derived macrophages (PDMs) within the lesion [8,10].

Anti-inflammatory cytokines, such as IL-10, can help reduce inflammation. For example, IL-10 has been shown to downregulate pro-inflammatory cytokines [11,12]. Some studies indicate that IL-10 promotes neuronal survival and functional recovery, while also alleviating neuropathic pain in SCI models [11,13,14].

After SCI, multiple factors simultaneously influence macrophages and microglia. Astrocytes become reactive, invade the area surrounding the lesion center, and contribute to the formation of a glial scar. This scar then limits recovery by acting as a physical and biochemical barrier to axonal regeneration [15,16].

Since multiple factors influence the progression of SCI, improving treatment effectiveness remains essential [17,18]. Although methylprednisolone has been extensively studied for acute SCI, its clinical use is limited due to side effects and uncertain effectiveness [19,20]. In contrast, several preclinical studies have investigated the therapeutic potential of estradiol (E2 or 17β-estradiol) in animal models of SCI. These studies show that E2 treatment can improve locomotor recovery after injury [21,22,23]. However, the effects of E2 depend on the dose, with high doses potentially causing unwanted side effects. In females, these side effects include gynecomastia, infertility, and an increased risk of hormone-sensitive cancers [24], while in males, excessive estrogen exposure has been linked to certain cancers and complications within androgen deprivation therapy [25].

Tibolone (Tib) is a synthetic steroid used in hormone replacement therapy and is also known for its neuroprotective properties [26]. Unlike estrogen, it does not exhibit estrogenic activity in endometrial or mammary tissues [27]. In the central nervous system (CNS), Tib reduces neuronal death, oxidative stress, and cognitive deficits in various animal models [27,28,29,30]. Additionally, in vitro studies on glial cells have shown that Tib decreases damage from oxidative stress and has anti-inflammatory effects by reducing the activity of nuclear factor κB (NF-κB) [31].

Previously, we evaluated the effect of Tib administration (2.5 mg/kg) over 21 days and found that this treatment significantly increased tissue preservation and improved motor function recovery after SCI [32]. However, we aimed to determine whether a shorter treatment duration could produce a recovery similar to that reported by Sanchez Torres et al. [32]. Additionally, previous studies have shown that Tib at a dose of 1 mg/kg improves systemic redox status and promotes neuroprotection [33,34].

On this basis, we evaluated the effects of Tib administered at 1 mg/kg (Tib 1) and 2.5 mg/kg (Tib 2.5) for 15 days on motor function recovery, inflammation, and gliosis in a rat model of SCI generated via moderate contusion.

## 2. Results

### 2.1. Tibolone Regulates Inflammation in Spinal Cord Injury

After SCI, proinflammatory cytokines such as IL-1β, TNF-α, and IFN-γ are quickly upregulated within hours, indicating an acute immune response [9,35]. Furthermore, serum levels of certain inflammatory cytokines remain elevated throughout the first week, contributing to secondary injury processes. By the second week after SCI, a transition toward chronic inflammation and potential immune regulation occurs [8,9]. Monitoring the levels of IL-1α, IL-1β, TNF-α, IFN-γ, GM-CSF, and IL-10 at 3 h, 3 days, 7 days, and 14 days post-SCI offers a detailed view of the inflammatory landscape. This approach helps identify key periods for immunomodulatory treatments and offers insight into how Tib affects SCI progression.

In a previous study, we assessed whether spinal cord injury (SCI) alters cytokine levels by comparing injured animals with an intact (non-injured) control group. We found that pro-inflammatory cytokines were significantly elevated three days post-injury relative to the intact group [36]. Notably, no differences were found between the non-injured group and the sham (laminectomy) group [36], consistent with previous studies [37]. Based on these findings, and to minimize the number of animals sacrificed, we opted not to include an intact control group in the present study. Instead, we incorporated a sham group that underwent the complete surgical procedure and postoperative care.

**Figure 1:**
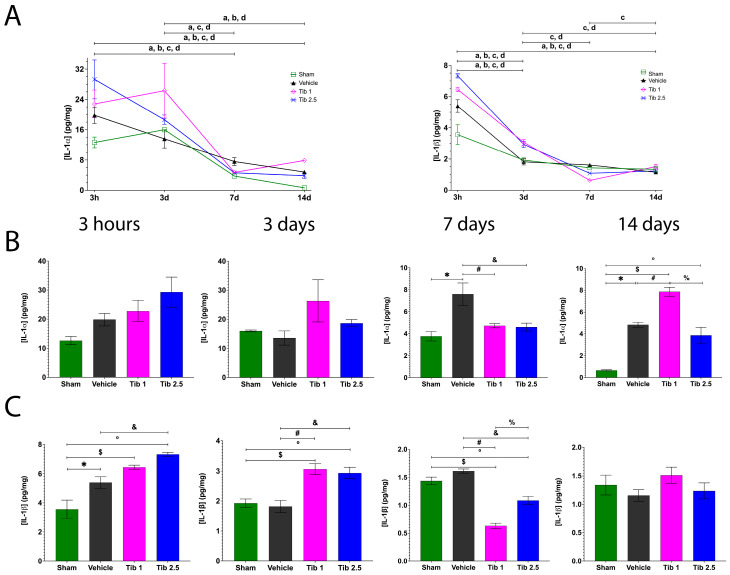
Concentrations of interleukin-1 alpha (IL-1α) and interleukin-1 beta (IL-1β) and the effect of Tibolone (Tib) after spinal cord injury (SCI). (**A**). Changes in IL-1α and IL-1β concentrations at 3 h, and at 3, 7, and 14 days post-surgery: Sham (□), Vehicle (**▲**), Tib 1 (◇), and Tib 2.5 (×). (**B**). Effect of Tib on IL-1α concentration (pg/mg) in spinal cord tissue at 3 h, and at 3, 7, and 14 days post-SCI. (**C**). Effect of Tib on IL-1β concentration (pg/mg) in spinal cord tissue at 3 h, and at 3, 7, and 14 days post-SCI. Experimental groups: Sham (laminectomy), Vehicle (SCI), Tib 1 (SCI treated with tibolone 1 mg/kg), and Tib 2.5 (SCI treated with tibolone 2.5 mg/kg). Data are expressed as the mean ± SEM (*n* = 6). Data were analyzed using one-way ANOVA, followed by the Tukey post hoc test (*p* < 0.05). Symbols indicate significant differences between groups: * Sham vs. vehicle; $ Sham vs. Tib 1; ° Sham vs. Tib 2.5; # Vehicle vs. Tib 1; & Vehicle vs. Tib 2.5; % Tib 1 vs. Tib 2.5. Letters indicate significant differences within each group across time points: a = Sham, b = Vehicle, c = Tib 1, and d = Tib 2.5.

**Figure 2:**
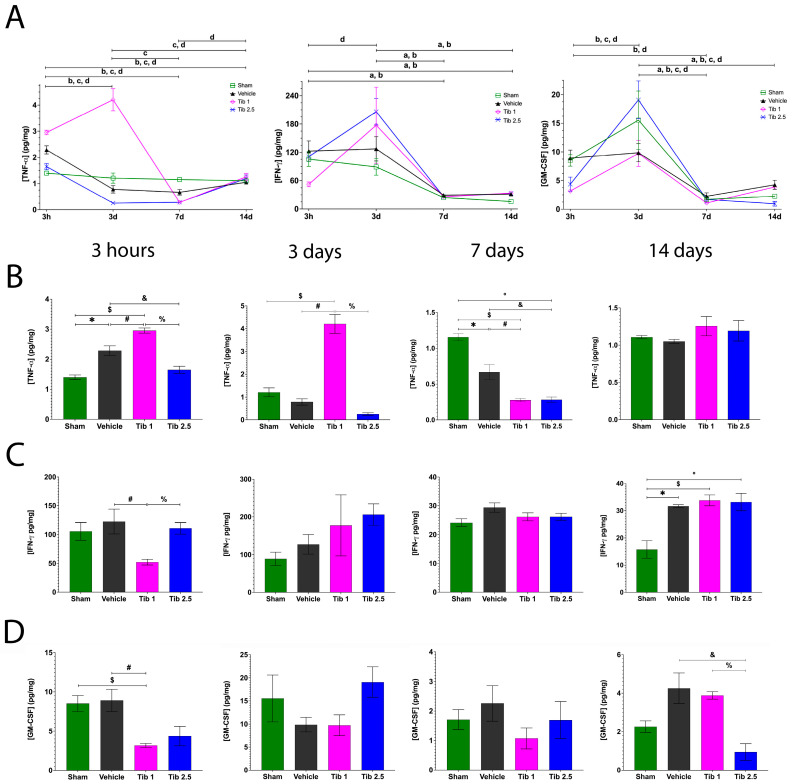
Concentrations of inflammatory cytokines and the effect of Tibolone (Tib) after spinal cord injury (SCI). (**A**). Changes in tumor necrosis factor-alpha (TNF-α), interferon-gamma (IFN-ɣ), and granulocyte macrophage colony-stimulating factor (GM-CSF) concentrations at 3 h, and at 3, 7, and 14 days post-surgery: Sham (□), Vehicle (**▲**), Tib 1 (◇), and Tib 2.5 (×). (**B**). Effect of Tib on TNF-α concentration (pg/mg) in spinal cord tissue at 3 h, and at 3, 7, and 14 days post-SCI. (**C**). Effect of Tib on IFN-ɣ concentration (pg/mg) in spinal cord tissue at 3 h, and at 3, 7, and 14 days post-SCI. (**D**). Effect of Tib on GM-CSF concentration (pg/mg) in spinal cord tissue at 3 h, and at 3, 7, and 14 days post-SCI. Experimental groups: Sham (laminectomy), Vehicle (SCI), Tib 1 (SCI treated with tibolone 1 mg/kg), and Tib 2.5 (SCI treated with tibolone 2.5 mg/kg). Data are expressed as the mean ± SEM (*n* = 6). Data were analyzed using one-way ANOVA, followed by the Tukey post hoc test (*p* < 0.05). Symbols indicate significant differences between groups: * Sham vs. vehicle; $ Sham vs. Tib 1; ° Sham vs. Tib 2.5; # Vehicle vs. Tib 1; & Vehicle vs. Tib 2.5; % Tib 1 vs. Tib 2.5. Letters indicate significant differences within each group across time points: a = Sham, b = Vehicle, c = Tib 1, and d = Tib 2.5.

**Figure 3:**
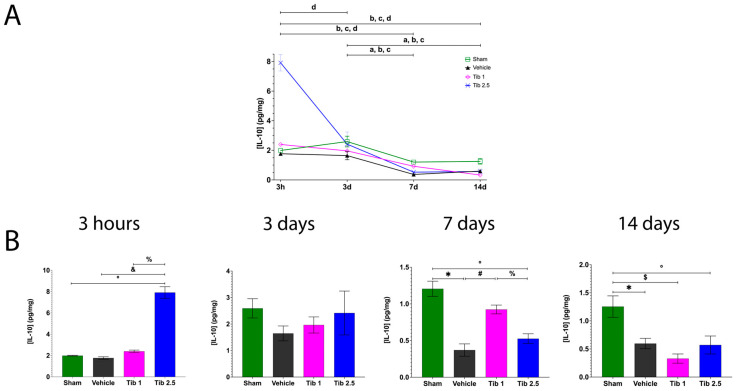
(**A**). Concentration of interleukin-10 (IL-10) and the effect of Tibolone (Tib) after spinal cord injury (SCI). (**B**). Changes in IL-10 concentrations at 3 h, and at 3, 7, and 14 days post-surgery: Sham (□), Vehicle (**▲**), Tib 1 (◇), and Tib 2.5 (×). (**B**). Effect of Tib on IL-10 concentration (pg/mg) in spinal cord tissue at 3 h, and at 3, 7, and 14 days post-SCI. Experimental groups: Sham (laminectomy), Vehicle (SCI), Tib 1 (SCI treated with tibolone 1 mg/kg), and Tib 2.5 (SCI treated with tibolone 2.5 mg/kg). Data are expressed as the mean ± SEM (*n* = 6). Data were analyzed using one-way ANOVA, followed by the Tukey post hoc test (*p* < 0.05). Symbols indicate significant differences between groups: * Sham vs. vehicle; $ Sham vs. Tib 1; ° Sham vs. Tib 2.5; # Vehicle vs. Tib 1; & Vehicle vs. Tib 2.5; % Tib 1 vs. Tib 2.5. Letters indicate significant differences within each group across time points: a = Sham, b = Vehicle, c = Tib 1, and d = Tib 2.5.

In this study, we measured the levels of IL-1α, IL-1β, TNF-α, IFN-γ, GM-CSF, and IL-10 in the spinal cords of rats from both the sham group and those with SCI treated with vehicle, Tibolone 1 mg/kg (Tib 1), or Tibolone 2.5 mg/kg (Tib 2.5) groups at 3 h and at 3, 7, and 14 days after SCI. Most cytokines reached their peak levels at 3 h post-SCI during the studied period (Figure 1, Figure 2 and Figure 3). IL-1α peaked at 3 h and decreased at 7 days and 14 days after surgery across all groups (Figure 1A). IL-1β also peaked at 3 h and decreased at 3 days, maintaining its levels through day 14 post-SCI across all groups (Figure 1A).

The effects of Tib 1 and Tib 2.5 on IL-1α and IL-1β levels at 3 h and at 3, 7, and 14 days post-SCI are shown in Figure 1B,C. There were no significant differences in IL-1α levels between the vehicle and Tib groups at both 3 h and 3 days post-SCI. However, IL-1α levels significantly decreased in the Tib 1 (4.72 ± 0.19) (*p* = 0.0143) and Tib 2.5 (4.60 ± 0.35) (*p* = 0.0115) groups compared to the vehicle group (7.59 ± 1.01) at 7 days post-SCI. Additionally, IL-1α concentration was significantly higher in the Tib 1 group (7.86 ± 0.40) than in both the vehicle (4.82 ± 0.22) and sham groups (0.65 ± 0.05) (*p* = 0.001) at 14 days post-SCI (Figure 1B). The concentration of IL-1β was significantly higher in the Tib 2.5 group (7.32 ± 0.14) than in the vehicle group (5.39 ± 0.40) (*p* = 0.0045) at 3 h post-SCI. Moreover, IL-1β levels increased in both the Tib 1 (*p* = 0.0091) and Tib 2.5 (*p* = 0.0151) groups at 3 days post-SCI but decreased significantly at 7 days post-SCI compared to the vehicle group (*p* < 0.0001 and *p* = 0.0007, respectively). Additionally, Tib administration led to significantly elevated IL-1β levels compared to the sham group at both 3 h and 3 days post-treatment. No significant differences were observed between the vehicle and Tib groups at 14 days post-SCI (Figure 1C).

Regarding TNF-α, the sham group maintained similar levels at all time points evaluated. TNF-α concentrations in the vehicle group increased at 3 h (2.28 ± 0.15), then decreased by day 3 (0.78 ± 0.14), and remained similar at days 7 (0.66 ± 0.10) and 14 (1.05 ± 0.02) post-SCI. The Tib 1 group showed the highest concentration at day 3 (4.20 ± 0.42) but decreased by day 7 (0.27 ± 0.02). TNF-α levels in the Tib 2.5 group at 3 h (1.64 ± 0.11) were similar to those in the sham group (1.39 ± 0.07) but decreased at days 3 (0.25 ± 0.05) and 7 (0.28 ± 0.03) post-SCI (Figure 2A).

The effects of Tib 1 and Tib 2.5 on TNF-α levels at 3 h and at 3, 7, and 14 days post-SCI are shown in Figure 2B. TNF-α levels were significantly higher in the Tib 1 group compared to the vehicle and sham groups at both 3 h and 3 days post-SCI. In contrast, TNF-α levels were significantly lower in the Tib 2.5 group (1.64 ± 0.11) compared to the vehicle group (2.28 ± 0.15) at 3 h (*p* = 0.0259) after SCI. Additionally, TNF-α levels decreased significantly in the Tib 2.5 group (1.64 ± 0.11 and 0.25 ± 0.05, respectively) compared to the Tib 1 group (2.95 ± 0.08 and 4.20 ± 0.42, respectively) at both 3 h and 3 days post-SCI (*p* < 0.0001). Furthermore, TNF-α levels were significantly lower in both the Tib 1 and Tib 2.5 groups (0.27 ± 0.02 and 0.28 ± 0.02, respectively) compared to the sham group (1.15 ± 0.05) and the vehicle group (0.66 ± 0.10) at 7 days post-SCI. No significant differences were observed among the groups at 14 days post-SCI (Figure 2B).

The levels of IFN-γ and GM-CSF significantly decreased from day 3 to day 7 across all groups (Figure 2A). Interestingly, Tib treatment altered the levels of IFN-γ at 3 h post-SCI; specifically, IFN-γ levels were significantly lower in the Tib 1 group (52.30 ± 4.99) than in the vehicle group (105.58 ± 15.47) (*p* = 0.0124). No other differences were observed among the groups at either 3 or 7 days post-SCI. Additionally, IFN-γ levels were significantly higher in the Tib 1 and Tib 2.5 groups (33.76 ± 1.99; 33.15 ± 3.19, respectively) compared to the sham group (15.77 ± 3.21) by day 14 post-SCI (Figure 2C).

Tib 1 treatment significantly lowered GM-CSF levels (3.18 ± 0.25) compared to both the vehicle (8.91 ± 1.41) and sham (8.52 ± 1.00) groups at 3 h post-SCI. No significant differences were found among the groups at both 3 and 7 days post-SCI. However, GM-CSF concentration was significantly lower in the Tib 2.5 group (0.94 ± 0.42) (*p* = 0.0081) compared to the vehicle (2.26 ± 0.30) and Tib 1 (3.88 ± 0.19) groups at 14 days post-SCI (Figure 2D).

Regarding the anti-inflammatory cytokine IL-10, its levels peaked at 3 h in both the sham and vehicle groups but gradually decreased from day 3 to 14 post-SCI. Additionally, the Tib 2.5 group showed the highest IL-10 level at 3 h, which then declined to levels similar to those of the other experimental groups (Figure 3A).

Figure 3B shows the effect of Tib 1 and Tib 2.5 on IL-10 levels at 3 h and at 3, 7, and 14 days post-SCI. The Tib 2.5 treatment increased IL-10 levels (7.91 ± 0.55) at 3 h compared to the sham (1.99 ± 0.03), vehicle (1.77 ± 0.11), and Tib 1 (2.39 ± 0.15) groups (*p* < 0.0001). Additionally, IL-10 levels were significantly higher in the Tib 1 group (0.9240 ± 0.66) compared to the vehicle group (0.3708 ± 0.008; *p* = 0.0036) at 7 days post-SCI. No significant differences were observed among the groups at 3 days post-SCI. However, IL-10 levels were significantly lower in both Tib groups compared to the sham group at 14 days post-SCI (Figure 3B).

### 2.2. Tibolone Regulates Gliosis in Spinal Cord Injury

The inflammatory response triggers rapid activation of astrocytes and microglia, leading to the release of proinflammatory mediators that contribute to secondary tissue damage [38,39]. This response begins within hours after SCI and can last up to three weeks, marking the subacute phase [40]. In rodent models of SCI, signs of astrogliosis appear as early as 2–7 days post-SCI [41,42]. By 14 days after SCI, microglia gather in the lesion and can be found between the fibrotic and glial scars, contacting astrocytes, where they remain for at least 35 days post-SCI [39].

**Figure 4:**
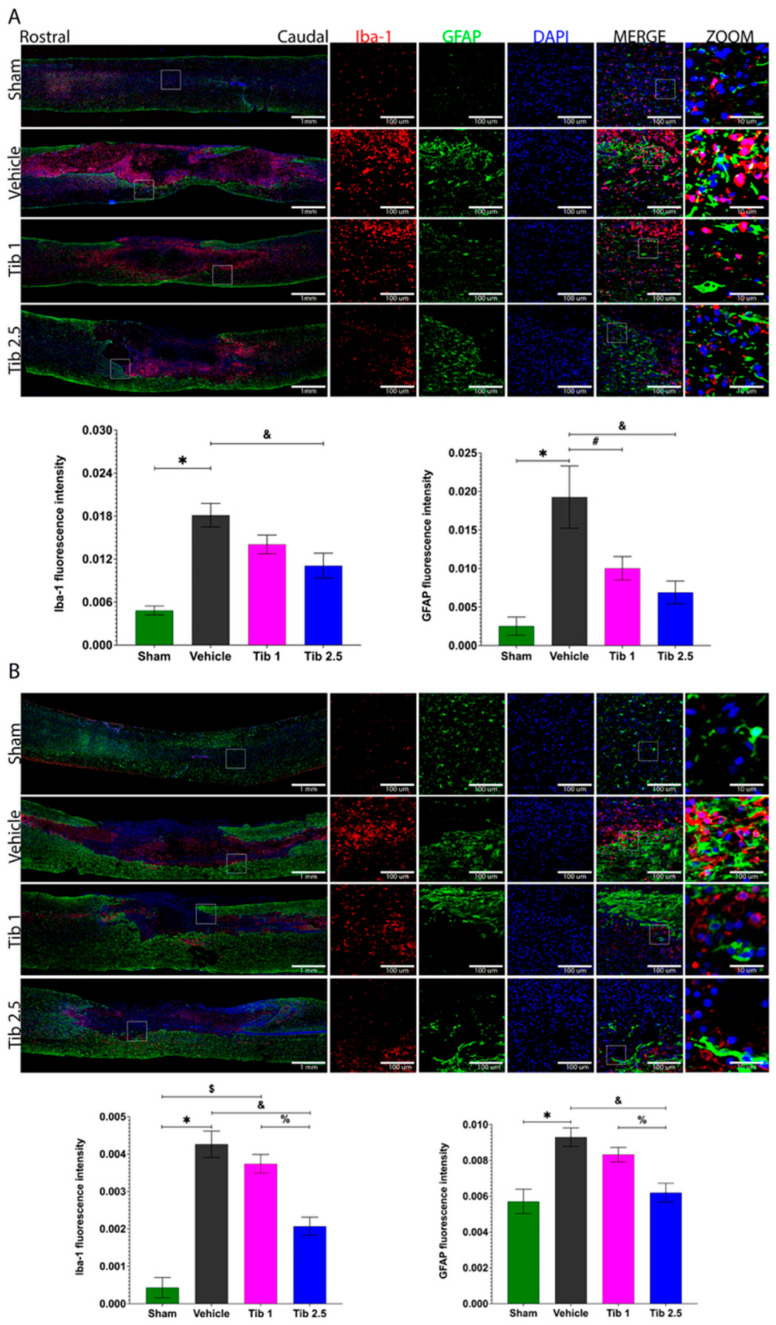
Tibolone reduced gliosis after spinal cord injury (SCI). (**A**). Immunohistochemical labeling of a representative longitudinal spinal cord section was performed at 7 days post-surgery for the Sham, Vehicle, Tib 1 (tibolone 1 mg/kg), and Tib 2.5 (tibolone 2.5 mg/kg) groups. The expression of ionized calcium-binding adaptor molecule 1 (Iba-1, microglia/macrophages) was labeled in red, glial fibrillary acidic protein (GFAP, astrocytes) in green, and nuclei (DAPI) in blue (scale bar = 100 μm). Quantitative analysis graphs of Iba-1 and GFAP (fluorescence intensity) expression in a 1 cm segment of the spinal cord, including the rostral region, the epicenter of the lesion, and the caudal region for each group in spinal cord sections. (**B**). Immunohistochemical labeling of a representative longitudinal spinal cord section was performed at 15 days post-surgery for the Sham, Vehicle, Tib 1 (tibolone 1 mg/kg), and Tib 2.5 (tibolone 2.5 mg/kg) groups. The expression of ionized calcium-binding adaptor molecule 1 (Iba-1, microglia/macrophages) was labeled in red, glial fibrillary acidic protein (GFAP, astrocytes) in green, and nuclei (DAPI) in blue. Scale bar = 100 μm, 10 μm (inset). Quantitative analysis graphs of Iba-1 and GFAP (fluorescence intensity) expression in a 1 cm segment of the spinal cord, including the rostral region, the epicenter of the lesion, and the caudal region for each group. Data are expressed as the mean ± SEM (*n* = 4). Data were analyzed using one-way ANOVA, followed by the Tukey post hoc test (*p* < 0.05). * Sham vs. vehicle; $ Sham vs. Tib 1; # Vehicle vs. Tib 1; & Vehicle vs. Tib 2.5; % Tib 1 vs. Tib 2.5.

We performed immunohistochemistry to quantify Iba-1 and GFAP expression and to assess whether Tib modulates gliosis at 7 days and 15 days post-SCI. Figure 4 shows that levels of Iba-1 and GFAP were higher in the vehicle group compared to the sham group at both time points. Although Iba-1 expression decreased with Tib 1 administration, the difference was not statistically significant. However, a significant decrease was observed with the Tib 2.5 treatment compared to the vehicle at 7 days (*p* = 0.0412) and 15 days post-SCI (*p* = 0.0167). Additionally, GFAP expression decreased with both Tib treatments, but statistical significance was only achieved with the Tib 2.5 treatment at 7 (*p* = 0.0106) and 15 days post-SCI (*p* = 0.0012) (Figure 4).

### 2.3. Tibolone Promotes Tissue Preservation

**Figure 5:**
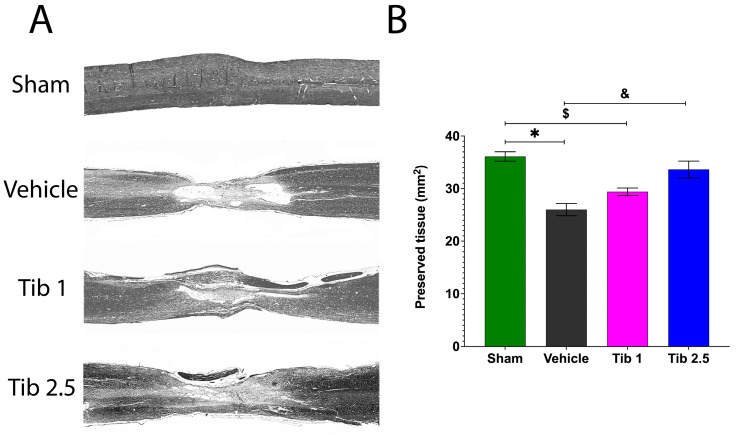
Tibolone administration preserved medullary tissue following spinal cord injury (SCI). (**A**). Representative images of longitudinal sections of the spinal cord stained with hematoxylin-eosin two months post-surgery: Sham, Vehicle, Tib 1 (tibolone 1 mg/kg), and Tib 2.5 (tibolone 2.5 mg/kg) groups. Images correspond to the rostral area, the epicenter of the injury, and the caudal area (panoramic 2× magnification). (**B**). Quantification of preserved tissue of a 2 cm segment of the spinal cord, including the rostral region, the epicenter of the lesion, and the caudal region, after 60 days of surgery in the sham, vehicle (SCI), or tibolone-treated (Tib 1 and Tib 2.5) animals. Data are presented as the mean ± SE area (mm^2^) of preserved tissue (*n* = 4). Data were analyzed using one-way ANOVA, followed by the Tukey post hoc test (*p* < 0.05). * Sham vs. vehicle; $ Sham vs. Tib 1; & Vehicle vs. Tib 2.5.

Sixty days after SCI, we performed a histological analysis to identify changes in the spinal cord’s cytoarchitecture (Figure 5). In the vehicle group, we observed an increase in the number of microcysts contributing to the extent of damage in both the rostral and caudal regions, along with cellular infiltration at the lesion epicenter, compared to the sham group. Unlike the vehicle group, animals treated with Tib mainly showed preserved tissue at the injury epicenter and less structural damage to nerve tissue in the rostral and caudal regions (Figure 5A). The area of preserved tissue decreased after SCI (26.03 ± 1.16) compared to the sham group (36.03 ± 0.89; *p* = 0.0001), while treatment with Tib at 2.5 mg/kg resulted in a significantly greater preserved tissue area (33.63 ± 1.63) compared to the vehicle group (*p* = 0.0028) (Figure 5B).

### 2.4. Tibolone Administration Improves Motor Function Recovery

**Figure 6:**
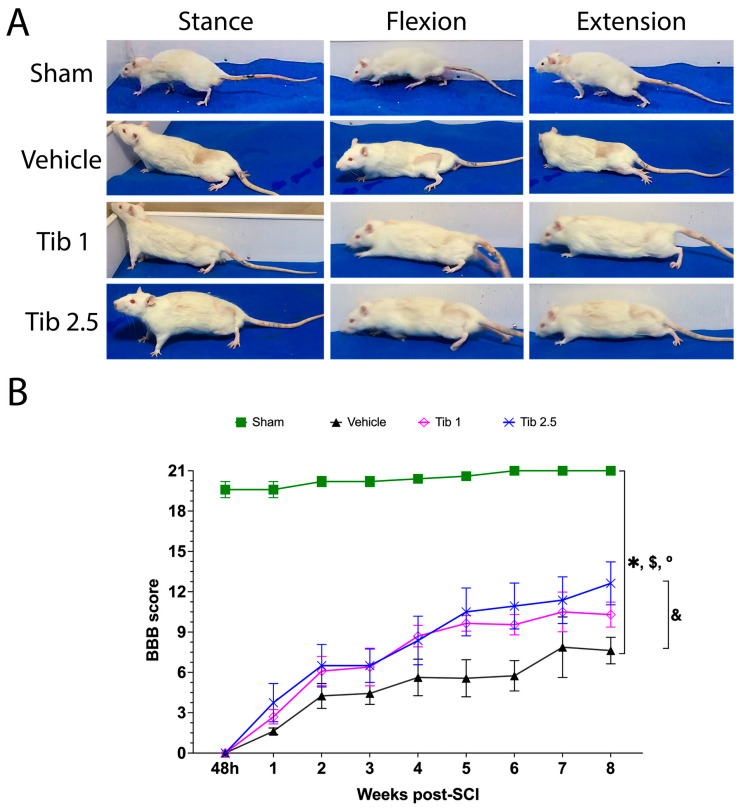
Tibolone effect on locomotor function recovery. (**A**). Representative and comparative images of animals from the Sham, Vehicle, Tib 1, and Tib 2.5 groups after 15 days of treatment and 8 weeks of open-field evaluation. (**B**). Locomotor function assessment using the Basso Beattie and Bresnahan (BBB) scale. Data are expressed as the mean ± SE for each group (*n* = 8). Data were analyzed using one-way ANOVA, followed by the Dunnett *post hoc* test (* *p* < 0.05). * Sham vs. vehicle; $ Sham vs. Tib 1; ° Sham vs. Tib 2.5; & Vehicle vs. Tib 2.5.

Motor function recovery was evaluated using the BBB scale. The sham group scored 19.6 at 48 h and reached the maximum score of 21 at six weeks post-laminectomy. The vehicle group achieved a final average BBB score of 7.62 ± 0.9, indicating that most animals had some mobility, though with limited or abnormal gait and movement patterns (Figure 6). The Tib 1 and Tib 2.5 groups posted final average BBB scores of 10.3 and 12.6, respectively. Notably, only treatment with Tib at a dose of 2.5 mg/kg produced a significant effect, while the 1 mg/kg dose showed only a trend toward improved functional recovery, as measured by the BBB scale. These scores reflect moderate to good recovery of motor function.

A BBB score of 10.3 shows that the animals can perform a few coordinated voluntary movements with their hind limbs and have improved stability. However, they still show changes in posture and gait, and sometimes in body weight support.

A BBB score of 12.6, achieved with Tib 2.5, indicates that the animals show significant recovery of motor function in the hind limbs, demonstrate frequent to consistent body weight support, and occasionally coordinate movement between the hind and forelimbs. Although their gait is not entirely normal, they can walk quite efficiently, showing some coordination and adequate support, albeit with minor changes in their movement patterns (Figure 6).

## 3. Discussion

Despite the promising therapeutic effects of estradiol, progesterone, and testosterone in SCI, their use is limited by side effects [43,44,45,46,47] and controversial results [48,49,50,51]. Consequently, some authors have proposed synthetic steroids that exert the neuroprotective effects of sex hormones without their adverse effects [44,45]. Tibolone (Tib) is a synthetic steroid considered a selective tissue estrogenic activity regulator (STEAR). Here, we evaluated the effect of Tib on neuroinflammation, gliosis, and motor function recovery in a rat model of incomplete SCI.

Tib has demonstrated anti-inflammatory, antioxidant, and neuroprotective effects. Furthermore, Tib has received approval from the Food and Drug Administration (FDA) for its use in alleviating adverse menopausal symptoms in women [52]. Additionally, Tib exhibits tissue-dependent activity, along with estrogenic, androgenic, or progestogenic effects [53,54]. As already mentioned, Sánchez-Torres et al. reported that Tib significantly increased the quantity of preserved tissue and enhanced motor function recovery after SCI [32].

In this study, we investigated the effect of a shorter treatment period with Tib (15 days). Additionally, we evaluated the effect of Tib at a dose of 1 mg/kg and 2.5 mg/kg on inflammation and gliosis in a rat model of SCI generated via moderate contusion.

E2 treatment decreases inflammation and enhances locomotor function after SCI in animal models [21,22,23,55,56,57]. Furthermore, E2 nanoparticles reduce the levels of inflammatory factors in plasma, cerebrospinal fluid, and spinal cord tissue, such as TNF-α, macrophage inflammatory protein-1 alpha (MIP-1α), IL-6, IL-4, IL-2, IL-10, IFN-γ, and other interleukins [45].

Akuzawa et al. found that IL-1α improved motor function recovery in a rabbit ischemic SCI model [58]. Additionally, IL-1 knockout was found to promote locomotor activity in a mouse transection SCI model [59], highlighting the importance of IL-1α inhibition in facilitating functional recovery. Our results showed that Tib treatments decreased IL-1α levels 7 days after spinal cord injury, along with improved motor function recovery.

In turn, IL-1β—one of the most extensively studied proinflammatory cytokines—impaired locomotion recovery in SCI models [60,61]. On one hand, we found that Tib treatments decreased IL-1β concentration 7 days post-SCI compared to the vehicle group. On the other hand, IL-1β levels increased in the Tib-treated groups 3 days post-SCI. The latter result is consistent with the effect of E2 observed by Ritz and Hausmann, who demonstrated that E2 treatment promotes an inflammatory environment by increasing IL-1α and IL-1β in the early acute phase of SCI [62]. Additionally, the Tib-induced increase in IL-1β levels observed 3 days post-SCI could promote IL-1 effects on neurogenesis, which have been previously demonstrated [63,64]. In this context, DeKosky et al. observed that administering an IL-1 receptor antagonist suppresses the nerve growth factor (NGF)-mediated reparative response in the CNS after trauma [65]. Moreover, Sato et al. observed that IL-1 participates in the classical and alternative activation of macrophages/microglia after SCI [59]. Furthermore, M2 macrophages/microglia can inhibit the immune response and promote repair [66]. Therefore, during periods that favor an increase in IL-1 concentration, Tib might promote repair after SCI through a similar mechanism.

Previous studies have shown that TNF-α impairs functional recovery in rat and mouse SCI models [67,68]. Additionally, TNF-α levels increase at the lesion epicenter following SCI [69], and TNF-α expression exacerbates cell death, edema, microvascular permeability, and lesion size [70,71]. Our results indicated that Tib modulated TNF-α levels in a concentration- and time-dependent manner. Tib 1 increased TNF-α concentration compared to the SCI group at 3 h and at 3 days post-SCI. In contrast, Tib 2.5 decreased this cytokine concentration compared to the SCI group at 3 h, 3 days, and 7 days post-SCI. Labombarda et al. demonstrated that P4 downregulated pro-inflammatory cytokines, including IL-1β, IL-6, and TNF-α, as well as enzymes involved in ROS production, such as COX-2 and iNOS, through a PR-dependent mechanism involving NF-κB inhibition [72]. On this basis, we suggest that this dual effect might be attributed to the progestogenic properties of Tib.

Several studies have highlighted the dual nature and complexity of IFN-γ in the context of SCI [73,74,75]. For instance, Fujiyoshi et al. reported that IFN-γ improved hind limb function in a mouse model of contusion SCI [73], whereas other researchers reported enhanced motor recovery in chimeric models lacking IFN-γ production [74], along with a reduction in macrophage polarization toward a proinflammatory M1 phenotype [75]. These findings suggest that both baseline functional status and the timing of IFN-γ intervention are critical determinants of therapeutic outcomes [74]. In our study, we observed that Tib modulated IFN-γ levels only at 3 h post-SCI, with Tib 1 treatment significantly decreasing IFN-γ concentrations compared to the vehicle group.

The effects of GM-CSF are controversial. Some authors have demonstrated that GM-CSF administration improves functional outcomes after rat-contusive SCI, increases brain-derived neurotrophic factor (BDNF) expression, stimulates axonal regeneration, and suppresses glial scar formation [76,77,78,79]. Other authors have shown that GM-CSF appears to be more relevant to tissue damage in CNS-driven inflammation by macrophages/microglia [80,81,82]. Our results showed that Tib 2.5 treatment decreased GM-CSF concentration at 3 h and 14 days post-SCI. Thus, Tib may have reduced GM-CSF’s pro-inflammatory mediators. Some studies have reported that this modulation could yield different outcomes. Parajuli et al. found that GM-CSF failed to induce TNF-α and NO in microglial activation [83]. However, Sisson et al. observed that a low dose of recombinant GM-CSF was sufficient to facilitate the production of TNF-α in human mononuclear cells [84]. In turn, Zhang et al. determined that GM-CSF administration induced the production of TNF-α and IL-1β by macrophages, thereby mediating CNS inflammation [82].

IL-10, an anti-inflammatory cytokine, promotes functional recovery in rats [13,85,86,87,88,89] and mice [90,91] following SCI. Additionally, IL-10 influences inflammation by activating microglia/macrophages [90,91] as well as astrocytes. Moreover, IL-10 enhances neuronal survival [87,92] and reduces cavitation [86] and tissue loss [93,94]. We found that a high dose of Tib increased IL-10 concentration at 3 h post-SCI, while a low dose of Tib elevated this cytokine concentration 7 days after the injury. This TIB-associated increase in IL-10 may relate to the effects observed in tissue preservation and functional recovery in the current results. These findings align with several authors who demonstrated the beneficial effect of IL-10 in promoting recovery after SCI [95,96] or are similar to the effects produced by some therapies for SCI, including exercise [97], transcranial direct current stimulation [98], and transplantation of mesenchymal stem cells [99], which stimulate the production of IL-10.

Once the spinal cord is injured, activated astrocytes and microglia form a dense border to isolate the severely damaged area [100]. Regulating reactive gliosis and neuroinflammation may represent a promising therapeutic approach for treating SCI. In vitro studies on glial cells have demonstrated that Tib reduces oxidative damage [101] and exhibits anti-inflammatory effects by decreasing NF-κB activation [31,102]. Tib reduced the number of glial fibrillary acidic protein (GFAP) immunoreactive astrocytes, the number of ionized calcium binding adaptor molecule 1 (Iba1) immunoreactive microglia, and the number of microglial cells exhibiting a reactive phenotype in the cerebral cortex of ovariectomized adult female mice 7 days after a stab wound brain injury, compared to vehicle-injected animals [103]. Similarly, our results indicate that Tib treatment reduces the number of GFAP+ astrocytes and Iba1+ microglia after SCI. These effects on gliosis may be linked to reduced neuronal loss, as shown by Sanchez-Torres et al., who observed beneficial homeostatic actions of Tib after SCI [32]. In the context of traumatic brain injury, gliosis and neuroinflammation are associated with delayed neuronal death that occurs several days after the primary neuronal loss induced by the injury [104].

Estradiol directly affects astrocytes through estrogen receptors (ERα and ERβ) [105,106,107,108,109], and can modulate astrocyte morphology and function via both nuclear and membrane-initiated estrogen receptor signaling pathways [110,111,112,113]. Notably, estrogen receptor expression is increased in reactive astrocytes compared to quiescent ones [114,115,116,117,118]. Additionally, some studies have shown that the effects of Tib on primary astrocytes, T98G cells, and BV-2 microglia are mediated by estrogen receptors (α or β) [31,102,119].

Evidence supporting the role of astrocytes in mediating estradiol’s neuroprotective effects in vivo comes from studies of conditional ERα knockout mice, where estrogen’s protective effects on neuroinflammation and astrogliosis were significantly reduced compared to wild-type animals [120]. The anti-inflammatory actions of estradiol on astrocytes through its receptors have been demonstrated in primary astrocyte cultures stimulated with lipopolysaccharide (LPS) and treated with selective ERα and ERβ agonists, specifically propyl pyrazole triol (PPT) and diarylpropionitrile (DPN), respectively. Treatment with the ERβ agonist DPN decreased the expression of inflammatory markers, including IL-1β, TNF-α, and MMP-9, while the ERα agonist PPT selectively decreased IL-1β levels. Considering that Tib effects could also be mediated by estrogen receptors, we suggest that Tib may exert anti-inflammatory and gliosis-modulating effects through estrogen receptor activation. However, further studies using selective silencing of ERα, ERβ, and tibolone treatment in SCI models are needed to confirm these mechanisms.

In addition, Tib may share similar properties with selective estrogen receptor modulators (SERMs) in regulating astrocyte-mediated neuroinflammation. Studies using primary astrocyte cultures exposed to inflammatory stimuli and treated with SERMs, such as tamoxifen, raloxifene, ospemifene, and bazedoxifene, have shown a reduction in the expression of inflammatory markers, including IL-6 and IP-10 [121]. The anti-inflammatory effects of SERMs in astrocytes are linked to the inhibition of the NF-κB signaling pathway. Beyond inflammation, SERMs mimic estradiol’s actions in other neuroprotective processes in astrocytes, including the regulation of glutamate transport, particularly GLT-1 expression through the CREB and NF-κB pathways [122,123,124,125], the promotion of neurotrophic factor release, such as TGF-β [126], and the modulation of aquaporin-4 expression [127]. Therefore, SERMs, including Tib, could be promising therapeutic options for promoting astrocyte-mediated neuroprotection, similar to estradiol. Furthermore, depending on its tissue-specific metabolism, Tib may activate androgen and progesterone receptors [128], which are also present in glial cells and influence gliosis [116,129,130,131,132,133,134]. Tib could potentially reduce reactive microglia by acting on androgen receptors, as these are known to decrease microglial activation after brain injury, at least in male rats [135]. Activation of progesterone receptors may also contribute to Tib’s effects on gliosis, as progesterone reduces inflammation and oxidative stress following traumatic brain injury [136,137].

Since SCI occurs more frequently in males [5,6], we chose to include only male rats in this study. However, this limitation became apparent in our research. Although some studies indicate that the progression and prognosis of SCI are not sex-dependent, increasing evidence suggests that sex can significantly influence many neurological diseases. Specifically, it has been demonstrated that astrocytes from males and females exhibit differences in their resistance to oxidative stress and oxygen–glucose deprivation [138]. Additionally, LPS stimulation results in higher levels of IL-6, TNF-α, and IL-1β in astrocytes from males or androgen-treated females compared to those from control or vehicle-treated females [139]. Furthermore, sex differences in astrocyte responses to estradiol, along with sex-specific hormonal effects on neurons and microglia [140,141,142], may explain the variability seen in estradiol’s neuroprotective effects [143,144].

Sexual dimorphism is also evident in how estradiol affects microglia. Interestingly, estradiol treatment produces anti-inflammatory effects in male microglia but induces a proinflammatory response in females. Conversely, in adult hippocampal microglia, estradiol exhibits a protective effect in females but not in males [142]. Additionally, estradiol modulates microglial phagocytic activity, decreasing it in females to levels comparable to those observed in males [145].

Furthermore, in vivo models of stress reveal sex-dependent patterns of microglial activation [146,147,148]. In vitro studies also demonstrate that male and female microglia exhibit distinct migratory behaviors, phagocytic capacities, and motility under both basal conditions and following treatment with IFN or LPS [142,149]. Notably, in neuropathic pain models, microglia play a key role in males, whereas they appear to be less involved in females [150,151,152,153]. Although several rodent studies have shown a neuroprotective benefit for females after SCI [154], other studies report no significant sex-related differences in functional recovery [155,156]. In any case, future research using female rats is critical to better understand potential sex-specific responses to SCI, ensure broader translational relevance, and assess the effects of Tib on SCI, including inflammation, gliosis, and functional recovery in females.

Based on the information described above, our findings showed that Tib regulated pro- and anti-inflammatory cytokine levels at 3 h, and at 3, 7, and 14 days post-SCI. Furthermore, Tib reduced GFAP and Iba-1 expression (gliosis markers), and promoted tissue preservation and functional recovery after SCI. Notably, Tib 2.5 showed the most pronounced and significant effects in reducing gliosis and promoting tissue preservation and functional recovery following SCI.

## 4. Materials and Methods

### 4.1. Animals

Adult male Sprague-Dawley rats, weighing 250–300 g, were housed under standard conditions (12-h light/dark cycles, 22 °C).

All surgical and experimental procedures were conducted in accordance with the regulations of the Mexican General Law of Health regarding research and science, as well as the Mexican Guidelines for Animal Care and Handling (NOM-062-ZOO-1999), with authorization from the National Committee for Scientific Research of the Mexican Institute of Social Security (registration number R-2015-785-060). Every effort was made to minimize animal discomfort and reduce the number of animals used.

### 4.2. Surgical Procedure

**Figure 7:**
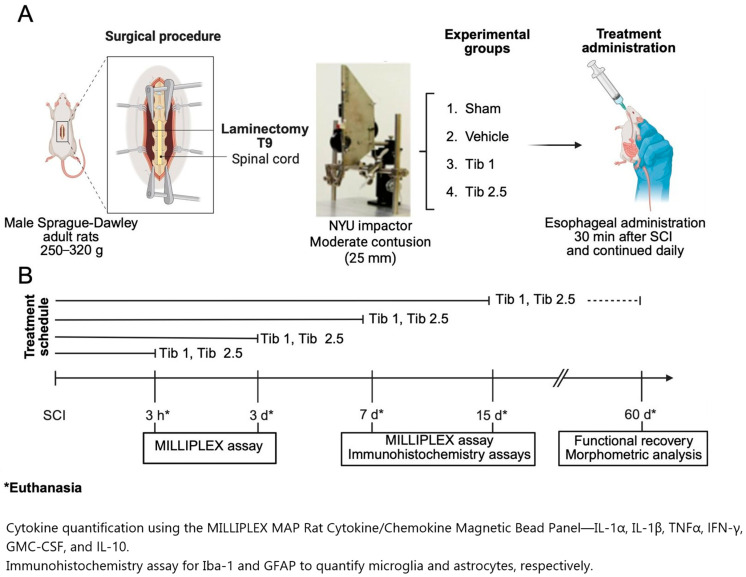
Schematic diagram of the experimental design. (**A**). Surgical procedure to induce moderate contusion spinal cord injury (SCI) at the thoracic vertebra 9 (T9) level. The animals were divided into four groups (Sham, Vehicle, Tib 1, and Tib 2.5) based on the treatments administered intragastrically via an esophageal cannula. (**B**). Timeline of the treatments following the analysis schedule. Sham: laminectomy; Vehicle: SCI; Tib 1: treated with tibolone 1 mg/kg; Tib 2.5: treated with tibolone 2.5 mg/kg. Cytokines IL-1α, IL-1β, TNF-α, GM-CSF, IFN-γ, and IL-10 were quantified using a MILLIPLEX MAP Rat Cytokine/Chemokine Magnetic Bead Panel–Immunology Multiplex Assay kit at 3 h, 3, 7, and 14 days post-surgery. Immunohistochemistry was used to measure Iba-1 and GFAP expression at 7 and 15 days post-SCI. Morphometric analysis and motor function recovery were assessed at 60 days post-surgery. Created with BioRender.com (accessed on 29 July 2025).

The animals were anesthetized intramuscularly using a mixture of Zoletil (20 mg/kg body weight) and xylazine (10 mg/kg body weight). Laminectomy was performed at the level of the thoracic 9 vertebrae (T9), following a weight-drop spinal cord contusion of moderate intensity induced by the NYU impactor device. This device consists of a 10 g impactor that was elevated (25 mm) above the surface of the spinal cord. Upon release, the impactor is accelerated by gravity until it strikes the exposed spinal cord. The contact surface of the impactor is round and slightly chamfered at the edges to prevent tearing of the dura mater. The diameter of the rod was 2.5 mm, slightly smaller than that of the spinal cord, allowing it to clear the edges of the vertebral canal as the impactor compresses the cord [157,158]. In the sham group, only a T9 laminectomy (without subsequent crush injury) was performed (Figure 7).

In the SCI groups, a hematoma at the lesion site was confirmed using a microscope. Subsequently, the muscle and skin were sutured in layers. All the animals received a single dose of 1,200,000 IU of benzathine penicillin intramuscularly, and an analgesic (paracetamol, 320 mg/L in water) was added to their drinking water for 5 days. The animals were housed individually in boxes in the vivarium under the conditions previously described. The bladder and intestine were manually emptied daily until the animals regained control of their sphincters. The surgical wound and the overall health of each animal were monitored daily.

### 4.3. Treatments

The animals were randomly divided into four groups as follows: Sham (laminectomy), Vehicle (SCI + vehicle), Tib 1 (SCI + Tib 1 mg/kg), and Tib 2.5 (SCI + Tib 2.5 mg/kg).

Animals from the SCI groups were administered tibolone (Tib) or a vehicle (water) orally, 30 min post-surgery, and then every 24 h, intragastrically via an esophageal cannula. Tib (Livial©, 2.5 mg tablets) was dissolved in water. Tib doses (1 mg/kg/day or 2.5 mg/kg/day) were selected based on previous studies that demonstrated Tib’s neuroprotective effects in improving memory and exhibiting antioxidant properties [26,27,28,29,30,32]. Administering Tib at these doses led to the upregulation of estrogen receptor expression. The activation of this receptor is linked to enhanced neuroprotection, as indicated by earlier studies that demonstrated the role of estrogen receptors in mediating neuroprotective effects following spinal cord and traumatic brain injury [159,160,161]. For cytokine concentration, animals from each group (*n* = 6) received treatment 30 min post-surgery and then every 24 h for 3, 7, and 14 days, depending on the experimental endpoint. For immunohistochemistry, animals from each group (*n* = 4) were treated for 7 and 15 days and euthanized thereafter. For motor function assessment, animals from each group (*n* = 8) were treated for 15 days and euthanized 60 days after SCI. A subset of these animals (*n* = 4) was used for morphometric analysis (Figure 7).

### 4.4. Cytokine Concentrations

For cytokine concentration analyses, trained personnel euthanized the animals via decapitation using a small animal guillotine (World Precision Instruments, Inc., Sarasota, FL, USA; Model DCAP-M, serial 133,708 9 K) in a room in which only one animal was placed at a time. Subsequently, spinal cord tissue (including the epicenter of the lesion plus 0.5 cm in the rostral direction and 0.5 cm in the caudal direction) was collected and preserved at 4 °C. Spinal cord tissues were homogenized with buffer (50 mM Tris-HCl pH = 7.5, 150 mM NaCl, 5 mM EDTA, 1 mM EGTA, 0.05% Tween 20, and Complete Protease Inhibitor (Roche)). The homogenate was centrifuged at 12,500 rpm for 30 min, and the supernatant was collected. Protein concentration was determined via the Bradford method (Quick Start Bradford 1× Dye Reagent, Bio-Rad, Hercules, CA, USA).

Cytokine quantification was performed at 3 h, 3 days, 7 days, and 14 days after the surgical procedure, using a MILLIPLEX MAP Rat Cytokine/Chemokine Magnetic Bead Panel–Immunology Multiplex Assay kit. Following the provider’s instructions, we measured the levels of IL-1α, IL-1β, TNF-α, GM-CSF, IFN-γ, and IL-10. The cytokine concentrations were normalized to the amount of protein in the samples and expressed in pg/mg of protein (Figure 7).

### 4.5. Immunohistochemistry

The immunohistochemistry assays were performed at 7 days and 15 days after the surgical procedure. The animals were anesthetized with pentobarbital and perfused intracardially with phosphate-buffered saline (PBS: 137 mM NaCl, 2.7 mM KCl, 10 mM Na_2_HPO_4_, 1.8 mM KH_2_PO_4_), followed by a 4% paraformaldehyde solution at a steady rate of 30 mL/min using a peristaltic pump. After perfusion, a 0.5 cm segment of the spinal cord was removed from the lesion center, and an additional 1 cm was retained from both the cephalic and caudal ends. Tissues were fixed in 4% paraformaldehyde for 8 days, then dehydrated through a graded series of alcohols (70%, 96%, and 100% ethanol), xylol, and embedded in paraffin. The tissues were embedded in paraffin blocks in a ventral–dorsal orientation. Spinal cord tissue sections (5 µm thick) were cut with a microtome RM2125 RTS (Leica Biosystems, Deer Park, IL, USA) and mounted on poly-L-lysine-coated slides. From each animal, slides were selected based on the location of the lesion epicenter and the ependymal canal.

The selected slides from each group (Sham, vehicle, Tib 1, and Tib 2.5) were dehydrated through a series of graded alcohols and then placed in a plastic Coplin jar containing 10 mM citrate buffer (pH 6) inside a pressure cooker for 20 min to recover the antigen. After this process, tissues were permeabilized with PBST (0.01 M PBS and 0.1% Triton) for 30 min, and nonspecific sites were blocked with 5% horse serum in PBST in a humid chamber for an additional 30 min. Tissues were incubated overnight at 4 °C in a humid chamber with rabbit anti-GFAP primary antibody (1:500, Cat. No. D4G40, Cell Signaling, Danvers, MA, USA) and rabbit anti-Iba1 primary antibody (1:500, Cat. No. D4G40, Cell Signaling, Danvers, MA, USA). Subsequently, tissues were washed with PBST and incubated with Alexa Fluor^®^ 488 donkey anti-rabbit secondary IgG antibody (1:500, Molecular Probes, Eugene, OR, USA) for 2 h at room temperature in the dark. The slides were then placed in a 0.1% solution of black Sudan B (Sigma-Aldrich, St. Louis, MO, USA) in 70% ethanol for 15 min, after which the excess black Sudan B was washed away with PBS. The tissues were covered with Vectashield (Vector Laboratories, CA, USA) and a coverslip. The slides were observed using the Aperio FL fluorescence scanner (Leica Biosystems, Pleasanton, CA, USA) at 1× magnification, and images were captured at 20× magnification. Specific fluorescence intensity was quantified in a 1 cm segment of the spinal cord, including the rostral region, the epicenter of the lesion, and the caudal region. Cells colocalizing with Iba-1-DAPI and GFAP-DAPI were quantified manually using FIJI software (NIH ImageJ, version 1.38×) (Figure 7).

### 4.6. Morphometric Analysis

The morphometric analysis was performed at eight weeks post-surgery. Spinal cord samples were taken from each group (sham, vehicle, Tib 1, and Tib 2.5) at the lesion epicenter to ensure consistent comparison across all study groups. The central canal (ependymal canal) served as a key reference point along the length of the spinal cord to achieve precise anatomical localization and reproducibility, with the spinal nerve roots used as additional landmarks to accurately locate the ependymal canal. Identification of the epicenter relied on visible injury features, such as cavitation, hemorrhage, and disruption of normal tissue structure, while vertebral landmarks guided standardization during the dissection process. Serial longitudinal sections of 5 µm thickness were cut with a microtome to ensure sample uniformity, selecting only slices that included the central spinal cord axis and clearly displayed the ependymal canal throughout their rostro-caudal length. Sections lacking a visible ependymal canal, exhibiting poor preservation, or showing significant artifacts like folds, tears, or incomplete mounting were excluded.

The slides per group were rehydrated through 1-min baths in paraffin, xylol, and a series of graded alcohols (100%, 95%, 85%, 70%, and 50% ethanol), followed by water. They were then stained with hematoxylin-eosin and covered with Entellan (Sigma Aldrich, St. Louis, MO, USA). Panoramic images were captured using a Leica Aperio CS2 brightfield scanner (Leica Biosystems, Deer Park, IL, USA) at 1× magnification. Additionally, photographs were taken at 20× magnification of a 2 cm segment of the spinal cord, including the rostral region, the lesion epicenter, and the caudal region. Fiji software (NIH ImageJ, version 1.38×) was used to measure the area (mm^2^) of preserved tissue (Figure 7).

### 4.7. Functional Recovery

Functional recovery was assessed using the Basso, Beattie, and Bresnahan (BBB) scale. The BBB scale evaluates the movement of three joints in the hindlimbs, plantar placement of the legs, support of body weight, and coordination between the forelimbs and hindlimbs in an open field. The scale ranges from 0, indicating no limb movement, to 21, indicating normal activity, with a maximum score of 21 points. Animals were observed for 4 min during each session in an open-field setting. Two independent, blinded observers performed the evaluation to ensure objectivity and reduce bias. The first assessment was conducted 48 h after SCI to confirm complete hindlimb paralysis. Afterwards, functional recovery was monitored weekly for eight weeks to track locomotor progress [162] (Figure 7).

### 4.8. Statistical Analysis

All data were analyzed and plotted using GraphPad Prism 5.0 software for Windows XP (Dotmatics, GraphPad Software, San Diego, CA, USA). The Levene’s test for homogeneity of variances was applied. Cytokine concentrations were assessed using one-way ANOVA, followed by Tukey’s multiple comparisons test, and presented as mean ± standard error (SE).

Preserved tissue areas were analyzed using one-way ANOVA, followed by Bonferroni’s post hoc test, and presented as mean ± SE. We considered *p*-values < 0.05 to be statistically significant. Data from BBB scores were analyzed using repeated-measures ANOVA, followed by Bonferroni’s post hoc test, and presented as mean ± SE.

## 5. Conclusions

Our results indicated that tibolone administration after spinal cord injury modulated neuroinflammation and altered the expression of GFAP and Iba-1 (gliosis markers), thereby preserving tissue and promoting motor function recovery. Although these effects occurred simultaneously, we cannot confirm a direct cause-and-effect relationship among them based on our data. Therefore, more research is necessary to determine if there are interconnected mechanisms that collectively enhance the beneficial effects of tibolone in SCI. The multiple actions of tibolone, however, strengthen its potential as a therapeutic option for patients with SCI and support further investigation.

## Data Availability

All data generated or analyzed during this study are included in this article.

## Abbreviations

The following abbreviations are used in this manuscript:

BDNF	brain-derived neurotrophic factor
CNS	central nervous system
DPN	diarylpropionitrile
GDNF	glial cell line-derived neurotrophic factor
GFAP	glial fibrillary acidic protein
GM-CSF	granulocyte-macrophage colony-stimulating factor
Iba1	ionized calcium binding adaptor molecule 1
IFN-γ	interferon-gamma
IGF-1	insulin-like growth factor 1
IL-1α	interleukin-1α
IL-1β	interleukin-1β
IL-6	interleukin-6
IL-10	interleukin-10
LPS	lipopolysaccharide
NF-κB	nuclear factor κB
PPT	propyl pyrazole triol
SCI	spinal cord injury
Tib	tibolone
TNFα	tumor necrosis factor-alpha
